# Longitudinal Changes in the Concentration of Major Human Milk Proteins in the First Six Months of Lactation and Their Effects on Infant Growth

**DOI:** 10.3390/nu13051476

**Published:** 2021-04-27

**Authors:** Jian Zhang, Ai Zhao, Shiyun Lai, Qingbin Yuan, Xiaojiang Jia, Peiyu Wang, Yumei Zhang

**Affiliations:** 1 Department of Nutrition and Food Hygiene, School of Public Health, Peking University, Beijing 100191, China; zhangjian92@pku.edu.cn; 2 Vanke School of Public Health, Tsinghua University, Beijing 100091, China; aizhao18@tsinghua.edu.cn; 3 Hangzhou Popide Sci. & Tech. Co., Ltd., Hangzhou 311112, China; laishiyun@126.com; 4 Junlebao Dairy Group, Shijiazhuang 050221, China; yuanqingbin@jlbry.cn (Q.Y.); xj_jia@126.com (X.J.); 5 Department of Social Medicine and Health Education, School of Public Health, Peking University, Beijing 100191, China; wpeiyu@bjmu.edu.cn

**Keywords:** human milk, protein, α-lactalbumin, casein, lactoferrin, osteopontin, infant growth

## Abstract

Our knowledge related to human milk proteins is still limited. The present study determined the changes in multiple human milk proteins during the first six months of lactation, investigated the influencing factors of milk proteins, and explored the impact of milk proteins on infant growth. A total of 105 lactating women and their full-term infants from China were prospectively surveyed in this research. Milk samples were collected at 1–5 days, 8–14 days, 1 month, and 6 months postpartum. Concentrations of total protein and α-lactalbumin were measured in all milk samples, and concentrations of lactoferrin, osteopontin, total casein, β-casein, α_s−1_ casein, and κ-casein were measured in milk from 51 individuals using ultra performance liquid chromatography coupled with mass spectrometry. The concentration of measured proteins in the milk decreased during the first six months of postpartum (*p*-trend < 0.001). Maternal age, mode of delivery, maternal education, and income impacted the longitudinal changes in milk proteins (*p*-interaction < 0.05). Concentrations of α_s−1_ casein in milk were inversely associated with the weight-for-age Z-scores of the infants (1 m: r −0.29, *p* 0.038; 6 m: r −0.33, *p* 0.020). In conclusion, the concentration of proteins in milk decreased over the first six months postpartum, potentially influenced by maternal demographic and delivery factors. Milk protein composition may influence infant weights.

## 1. Introduction

Human milk (HM) is the most optimal food for infants. Both the World Health Organization (WHO) and the United Nations Children’s Fund recommend exclusive breastfeeding in the first six months of life to achieve optimal growth and development [1,2]. In recent decades, the nutritional value of HM has been extensively investigated. Studies revealed that breastfeeding has some short-term and long-term benefits for infants, such as reduced risks of illness, obesity, and diabetes [3,4,5,6]. Studies showed that formula-fed infants had different growth patterns compared to breastfed infants [3,7,8]. 

It is widely recognized that the proteins in HM are one of the major contributors to the beneficial effects of HM on infant growth and development [7,9]. Proteins are the third largest macronutrient in HM [10]. These proteins provide not only essential nitrogen and amino acids required for growth but also bioactive proteins and peptides with special functions [10]. The HM proteome is complex, and about 2500 different species have been identified [11]. α-lactalbumin, lactoferrin, serum albumin, and caseins are the most abundant species, making up about 85% of the total HM proteins [12]. α-lactalbumin is the most abundant whey protein [13] and provides infants with essential amino acids, making it possible to lower the total protein content when added to infant formulas. α-lactalbumin is reported to support appropriate growth [14] with increased energetic efficiency [15] and good gastrointestinal tolerance [16]. Lactoferrin is the second most abundant whey protein in HM [17] and is known particularly for its bacteriostatic activities [3,17,18]. Caseins are thought to be of predominantly nutritional value because they are completely digested in infants’ intestines [19]. Osteopontin is another protein that has received considerable attention in recent years. It was reported that osteopontin might influence the metabolism of amino acids and cytokine responses in formula-fed infants [20]. Animal experiments suggested that osteopontine could promote intestinal and brain development in early life [21,22]. Moreover, a study showed that osteopontin plays a role in bone metabolism and homeostasis [23].

The contents of proteins in HM are dynamic during the lactation period and are reported to be related to factors from mothers, infants, and the environment [24]. These changes are thought to be important as proteins play significant roles in infant health [19], and these changes may reflect the interactions between mothers, infants, and the environment. Depicting the longitudinal patterns of milk proteins also provides useful information for the manufacture of infant formula, which is an alternative for infants who cannot obtain enough HM. To date, some protein components in HM have been widely investigated, such as total protein [25] and lactoferrin [17]. Meanwhile, data on some other components (e.g., osteopontin and casein fractions) remain scarce. Previous studies suggested that there might be differences in some components in milk from Chinese women and that from other populations, such as lactoferrin [17,26] and polyunsaturated fatty acid [27]. However, our knowledge on the longitudinal changes of major HM proteins in Chinese lactating women is limited. Moreover, although HM has been extensively investigated in recent decades, evidence about HM protein in relation to the growth of infants is currently limited in the literature [8,28]. 

The present study aimed to depict the longitudinal changes of total protein, α-lactalbumin, lactoferrin, osteopontin, and caseins (total casein, β-casein, α_s-1_ casein, and κ-casein) in milk from Chinese lactating women during the first six months of lactation. Furthermore, we investigated the effects of general characteristics on the concentrations of proteins and the association of milk proteins with the growth of infants.

## 2. Materials and Methods

### 2.1. Study Design and Surveys

The present research utilized a cohort study to understand the HM composition and association of HM to the growth of infants. Women in the third trimester of pregnancy were introduced to this project, and those who agreed to participate were registered and further enrolled in the survey. The enrollment was conducted in three hospitals located in Beijing, Suzhou, and Xuchang in China, and the survey was conducted from December 2017 to December 2018. Eligibility criteria included women aged 18 years and above, singleton pregnancy, and with intention to breastfeed for 4 to 6 months postpartum. Exclusion criteria included smoking, drinking, diabetes, hypertension, infectious diseases, and preterm birth (gestation <37 weeks). The baseline surveys were conducted at 1–5 days postpartum at the participants’ homes. Interviewer-administered questionnaires were used to collect baseline information, including sociodemographic characteristics (age, education, and household income), medical history (history of pregnancy and delivery), and neonatal and delivery information (gestational age at birth, mode of delivery, and infant gender). Follow-up surveys were conducted at 8–14 days, 1 month, and 6 months during lactation in the hospital. All the follow-up surveys were scheduled to coincide with postnatal assessment or the children’s vaccination schedule. Trained college students and nurses finished all the interviews in the survey. A total of 105 participants with three or more milk samples were included in the present study. This study is exploratory research, and the sample size was determined according to the resources that the research team obtained. The concentrations of total protein and α-lactalbumin in the milk samples from all the participants were measured, while the concentrations of other indices were only tested for 51 participants due to restrictions of funding.

### 2.2. Human Milk Collection

Milk samples were collected at 1–5 days (colostrum), 8–14 days (transitional milk), 1 month (early mature milk), and 6 months (mature milk) postpartum. All the samples were collected in the morning. On the survey days, participants were asked to feed their children before 7 a.m., and milk samples were collected between 9 a.m. and 11 a.m. We had no restriction on participants’ diet before sample collection. First, the breast on one side was cleaned using clean water and wiped with sterile gauze. Second, previously trained nurses squeezed the milk of a single breast into a sterile bottle. The bottle was then inverted for 5–6 times to mix the milk by hand, and a 40 mL sample (15 mL for colostrum and transitional milk) was retained for further analysis. The remainder of the milk was returned to the mothers for infant feeding. Hand expression by trained research personnel is preferable. However, pump expression or hand expression by the mother herself were offered as alternatives for the convenience of the participants. Samples were stored at −20 °C in the hospital for 1 week maximum before transfer to the laboratory in Peking University. Third, after transfer to the laboratory, each milk sample was distributed in 1 mL freezing tubes and stored at −80 °C.

### 2.3. Human Milk Measurements

The concentration of total protein in the milk samples was determined by the Bradford method [29]. The Coomassie Blue reagent was prepared by dissolving 5 mg of Coomassie Brilliant Blue G 250 in 25 mL of ethanol (95%) and by adding 50 mL of phosphoric acid (88%) and 425 mL ultrapure water. This was followed by filtration. Then, 0.1 g milk samples were diluted to 10 mL with water, and 5 μL of diluted milk and 200 μL Coomassie Blue reagent were mixed in a 96-well plate. The percentage of transmittance at 595 nm was recorded after two minutes. The standard curve was generated using bovine serum albumin.

Concentrations of α-lactalbumin, lactoferrin, β-casein, α_s−1_ casein, and κ-casein in the milk samples were quantified using the protocol adapted from a previously published method [30]. Each 0.2 g milk sample was diluted to 10 mL with ultrapure water. An aliquot of 10 μL of a diluted milk sample was mixed with 10 μL of an internal standard, 10 μL of dithiothreitol solution (15 mg/mL water), and 845 μL of water. The internal standards were isotope-labeled peptides purchased from ChinaPeptides Co. Ltd. (Shanghai, China). Details of the internal standards are shown in Table S1. The mixture was then incubated in an 80 °C water bath for 30 min. After that, 10 μL of iodoacetamide solution (54 mg/mL water) was added, and the mixture was left to react at room temperature in the dark for 30 min. For digestion, the mixture was added to 10 μL of trypsin solution (0.4 mg/mL in 1 mmol/L hydrogen chloride) and 100 μL of ammonium bicarbonate solution (39.6 mg/mL in water) and allowed to react in a 37 °C water bath for 4 h. Then, 5 μL of formic acid was added to terminate the digestion. The mixture was homogenized and filtrated through a 0.22 μm nylon filter before further analysis. For quantification, peptide samples were analyzed using Acquity ultra-performance liquid chromatography (I-CLASS) coupled with a triple quadrupole mass spectrometer equipped with an electrospray ion source in multiple reaction monitoring modes (Waters, Milford, MA, USA). An Acquity BEH300 C18 column (1.7 μm particle size, 2.1 × 100 mm) was used. Peptides were separated using an 8-min binary gradient consisting of solvent A (water with 0.1% formic acid) and solvent B (acetonitrile with 0.1% formic acid) at a flow rate of 0.3 mL/minute. The elution program started by increasing 3% B to 32% B over 5 min, ramping up to 100% B over 0.1 min, holding at 100% B for 1 min, decreasing to 3% B over 0.1 min, and then holding at 3% B for 1.8 min. The column temperature was 40 °C. The conditions of the mass spectrometer were set as follows: ionization mode, ESI+; capillary voltage, 3.5 kV; source temperature, 150 °C; desolvation temperature, 500 °C; cone gas flow, 150 L/h; desolvation gas flow, 800 L/h; and argon collision gas pressure, 3 × 10^−3^ mbar. The standard curve was generated by using protein-specific signature peptides. The concentration of total casein was the sum of the concentrations of β-casein, α_s-1_ casein, and κ-casein.

For osteopontin in the milk samples, each 0.5 g of the milk sample was diluted to 10 mL with ultrapure water. One-hundred microliters of the diluted milk sample were then mixed with 50 μL of an internal standard (ChinaPeptides Co. Ltd., Shanghai, China), 780 mL of sodium bicarbonate solution (100 mmol/L), and 10 μL of dithiothreitol solution (500 mmol/L), which was followed by incubation in a 70 °C water bath for 30 min. After cooling to room temperature, 30 μL of iodoacetamide solution (500 mmol/L) was added to react for 30 min in the dark. For digestion, 20 μL of trypsin solution (1 mg/mL) was added. After overnight digestion at 37 °C, the process was terminated by adding 10 μL of formic acid. After homogenization, the solution was filtrated through a 0.22 μm of nylon filter before further analysis. The same ultra-performance liquid chromatography system, column, and mobile phases were used for quantification. For elution of the peptides, solvent B was held at 5% for 1 min, increased from 5% to 50% over 4.8 min, ramped up to 100% over 0.2 min, held at 100% for 0.8 min, decreased to 5% over 0.2 min, and then held at 5% for 2 min. The column temperature was 35 °C. Details of the triple quadrupole mass spectrometer conditions were as follows: working in multiple reaction monitoring modes: ionization mode, ESI+; capillary voltage, 4.5 kV; capillary temperature, 325 °C; desolvation temperature, 375 °C; desolvation gas flow, 11.5 L/min; and sheath gas flow rate, 10 L/min.

All samples were analyzed in duplicate. We ran a quality control test every day, and the quality control data were shown in Table S2. The concentrations of HM proteins were expressed as g/100 g. To be consistent with the data in the literature, the concentrations were converted to g/100 mL. When converting, the HM density was regarded as 103.2 g/100 mL [25].

### 2.4. Anthropometric Measurements

Maternal weight before pregnancy was based on self-reporting, and height was measured to the nearest 0.1 cm without shoes using a portable height measuring instrument at 1 month postpartum. Body mass index (BMI) was calculated as weight (kg)/(height(m))^2^. Pre-gestational BMI was converted into a categorical variable based on the median (<20.7 or ≥20.7 kg/m^2^). At 1 month and 6 months postpartum, the lengths and weights of the infants were measured by Infant/Child ShorrBoard. Z-scores of the length-for-age, weight-for-age, and weight-for-length were calculated according to the WHO Child Growth Standards [31].

### 2.5. Statistics

Results are presented as the means and standard deviations for continuous variables and percentages for categorical variables. Categorical variables were compared using Chi-square tests between groups.

Longitudinal changes in milk protein concentrations across lactation periods were analyzed using the linear mixed effect models, with postpartum time (weeks) as a fixed effect and the participant as a random intercept. Differences between surveys (1–5 d vs. 8–14 d, 8–14 d vs. 1 m, and 1 vs. 6 m) were analyzed using one-way repeated measures ANOVA tests, with the concentration of proteins as the repeated measure and postpartum time as the independent variable.

The overall effects of baseline characteristics on the concentrations of milk proteins were investigated with the linear mixed effects models. Each model included the fixed effects of postpartum time (weeks), a predictor (maternal age, education, household income, pre-gestational BMI, mode of delivery, parity, and gender of infant), and an interaction item for postpartum time and the predictor, as well as a random intercept for each participant. Differences in milk protein concentrations across baseline characteristics at each time point were compared using Student’s t-test.

The Z-scores of the anthropometric indices of infants between surveys were compared using one-way repeated measures ANOVA tests. The associations between concentrations of milk proteins and the anthropometric indices of infants were analyzed via Pearson correlation. The cumulative average concentrations of milk proteins before anthropometric measurements were calculated to represent long-term exposure, e.g., when estimating the correlation between α-lactalbumin and the length of the infant at 1 month postpartum, the average concentrations of α-lactalbumin measured at 1–5 and 8–14 days postpartum were calculated and subsequently used in the analysis. 

Before analysis, the values of lactoferrin concentration in milk were transformed to the log scale to increase normality. No imputation method was applied for missing data. All the statistics were analyzed in R 4.0.3 (R Core Team, Vienna, Austria). Additional packages were used for plots (corrplot [32]), calculating Z-scores of the anthropometric indices of the infants (anthro [33]), and linear mixed effects models (lme4 [34] and lmerTest [35]). All *p* values were two-sided, and statistical significance was defined as *p* < 0.05.

## 3. Results

### 3.1. Subject Characteristics

General characteristics of the lactating mothers and corresponding infants are shown in Table 1. Milk samples from all the 105 participants were tested for concentrations of total protein and α-lactalbumin, and samples from 51 participants were additionally tested for other proteins (lactoferrin, osteopontin, β-casein, α_s-1_ casein, and κ-casein).

### 3.2. Contents of Protein Components in Human Milk

The concentration of total protein in the milk decreased gradually during the first six months of lactation (Table 2). When compared at the same time points, women aged 30 years and above had a higher concentration of total protein at 6 months postpartum. Women with a college degree and above had a higher concentration of total protein in their milk at one month postpartum (Table S3).

The concentration of α-lactalbumin remained relatively stable during the first two weeks postpartum and then decreased slightly in the first month (Table 2). A higher concentration of α-lactalbumin in the colostrum was found to be related to higher maternal education. Significant interactions were observed between postpartum time and maternal education and income (Table S3).

The concentration of lactoferrin decreased sharply during lactation (Table 2). Women with a higher pre-gestational BMI had a higher concentration of lactoferrin at one month postpartum. Women who had a Cesarean delivery featured higher concentrations of lactoferrin than those who experienced vaginal delivery at six months postpartum. We found a significant interaction between postpartum time and mode of delivery in relation to the concentration of lactoferrin (Table S3).

The concentration of osteopontin showed a downward trend across lactation (Table 2). A higher maternal age and maternal education were associated with a higher concentration of osteopontin at six months postpartum. Women who gave their first birth had a higher concentration of osteopontin at one month postpartum. A significant interaction was observed between postpartum time and maternal age in relation to the concentration of osteopontin (Table S3).

Collectively, total casein was the most abundant protein in milk during the first six months of lactation. Among casein fractions, β-casein was the most abundant component, followed by α_s-1_ casein and κ-casein. Systematically, the concentration of β-casein decreased during lactation, but the difference between colostrum and transitional milk was insignificant (Table 2). The concentrations of α_s-1_ casein and κ-casein decreased across the lactation periods (Table 2). Women aged 30 years and above had higher concentrations of total casein at six months postpartum and higher α_s-1_ casein in milk at 8–14 days and six months postpartum. Women with a higher pre-gestational BMI had a higher concentration of α_s-1_ casein at one month postpartum. Women who experienced vaginal delivery had a higher concentration of total casein and β-casein at 1–5 days and a lower concentration of κ-casein at six months postpartum. Significant interactions with postpartum time were found for mode of delivery in relation to total casein and β-casein (Table S3).

**Figure 1:**
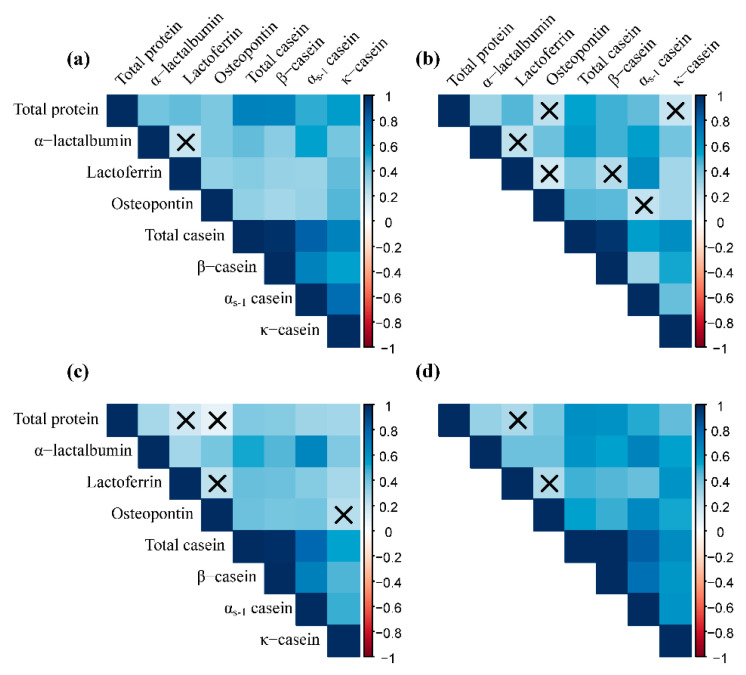
Correlation between human milk proteins. × *p* ≥ 0.05. The color in the square represents the value of the correlation coefficient. Samples with concentration information on all eight human milk indices were included in the analysis (number of participants: 51). Correlation coefficients were calculated by Pearson correlation. The values of lactoferrin concentration were transformed to log scale before analysis: (**a**) 1–5 days postpartum, (**b**) 8–14 days postpartum, (**c**) 1 month postpartum, and (**d**) six months postpartum.

The correlation between milk proteins is shown in Figure 1. The concentrations of most milk proteins were positively correlated with each other in most surveys.

### 3.3. Concentrations of Human Milk Proteins and Growth of Infants

**Figure 2:**
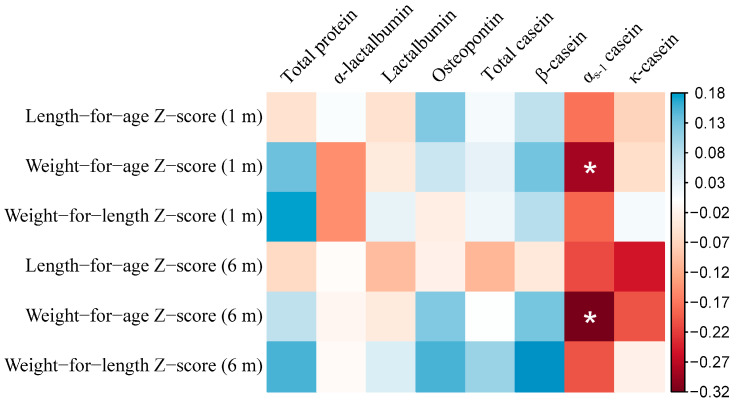
Correlation between human milk proteins and the growth of infants. * *p* < 0.05. The color in the square represents the value of the correlation coefficient. The concentrations of total protein and α-lactalbumin in the milk samples from all the participants were measured, while the concentrations of other indices were only tested for 51 participants in the subgroup. Correlation coefficients and *p* values were estimated via Pearson correlation. The values of lactoferrin concentration were transformed to log scale before analysis. The cumulative average concentrations of milk proteins before anthropometric measurements were calculated to represent long-term exposure, e.g., when estimating the correlation between α-lactalbumin and the length of the infant at one month postpartum, the average concentrations of α-lactalbumin measured at 1–5 and 8–14 days postpartum were calculated, and, subsequently, used in the analysis.

The anthropometric characteristics of the infants during follow-up are shown in Table 3. The length-for-age Z-score at six months postpartum was higher than that at one month postpartum. The cumulative average concentration of total protein tended to be positively correlated with weight-for-length Z-score at one month postpartum (*p* 0.069). Average α_s-1_ casein was inversely associated with the weight-for-age Z-score at one month and six months postpartum (Figure 2).

## 4. Discussion

The present study determined the concentrations of major milk proteins during lactation using milk samples from Chinese lactating women. The results contribute to our knowledge of human milk proteins, especially osteopontin and casein fractions. This study also prospectively investigated the impacts of milk proteins on infant growth. To the best of our knowledge, our study is one of the first to depict the longitudinal patterns of multiple milk proteins in Chinese women over a long lactation period.

In the study population, the concentration of total protein in milk decreased gradually across the first six months of lactation. Both the trend and concentration of total protein were close to those in the data from China in the literature [36,37]. The concentration of total protein in mature milk in this study was similar to that in other populations [38,39], but the value in colostrum was lower in milk from Chinese mothers compared to that in milk from Turkish and Swiss mothers [40,41]. α-lactalbumin is the predominant whey protein in HM. The concentrations of α-lactalbumin in milk from different populations have been reported, including Switzerland [9], the United States [19], Malawi [42], and China [13,36]. The present study found the concentration of α-lactalbumin remained relatively consistent in the first two weeks of lactation and then decreased slightly in the first month postpartum. The trends were similar to those from a cross-sectional study in China, which showed that the concentrations of α-lactalbumin at 5–11 days and 12–30 days postpartum were similar [13]. However, the concentration of α-lactalbumin in the present study was lower than that in milk from Swiss and American women [9,19]. The concentration of lactoferrin in HM has been relatively widely investigated [17]. In this study, the concentration of lactoferrin declined sharply during lactation, which was consistent with the literature, but the concentration of lactoferrin in milk from Chinese women was lower than the global mean [17]. It was worth noting that the inter-individual variability of lactoferrin decreased with an increase in lactation. Osteopontin has been a research hotspot in recent years. However, data on osteopontin in HM are limited. Goonatilleke et al. reported that the concentration of osteopontin in milk from the colostrum in American women increased until week 2 and then decreased [19]. However, using milk from American women, Jiang et al. reported that the osteopontin concentration was high on day 1 to 8 and decreased after day 9 [43]. Our study revealed that the concentration of osteopontin decreased gradually in milk from Chinese women during lactation, but the concentration of osteopontin was higher than that in the data published by Jiang et al. [43] and in the data on Danish lactating women [44].

Caseins are a group of proteins that differ in their chains of amino acids and structures. According to the literature, a total of three kinds of caseins (β-casein, α_s-1_ casein, and κ-casein) were identified in HM [18,45]. Although the concentrations of total casein in milk from several populations have to be reported [9,13,46], casein fractions, especially α_s-1_ casein and κ-casein, were seldom investigated. Our study found that all casein fractions decreased in milk from Chinese women during lactation. β-casein was found to be the most abundant casein fraction in the present study, and the concentration of β-casein in this study was close to that in the data from China [36], but higher than that in the data from American and French women [19,47]. It is recognized that κ-casein is the second most abundant casein in HM. However, we found that the concentration of κ-casein was lower than that of α_s-1_ casein in milk from Chinese women. The concentration of κ-casein was similar to that in previously published data on Chinese milk [36], but much lower than that in the data on American and French milk [19,47]. In our study, the concentration of α_s-1_ casein was about twice as great as the concentration of κ-casein.

Although the beneficial effects of HM have been extensively investigated in recent years, evidence on HM proteins in relation to infant growth is sparse, especially for term infants [8,28]. Prentice et al. reported that protein in hindmilk at 4–8 weeks postpartum can be positively associated with the BMI of infants (gestation ≥36 weeks) at 12 months [48]. Our study found that the concentration of total protein in milk tends to be related to the weight-for-length Z-scores of term infants at one month postpartum (r 0.18, *p* 0.069). This association seemed to be reasonable, as proteins provide infants with nitrogen and amino acids, which are essential for infant growth. However, the mechanisms of how HM proteins impact infant growth are complicated. To date, evidence on the protein intake and growth of infants has mainly come from studies on formula. The early protein hypothesis suggests that a higher protein intake from infant formulas may increase branch-chain amino acids, which may stimulate the secretion of insulin and insulin-like growth factor 1 (IGF-1) in infants. These factors may promote growth and adipogenic activity [49,50]. However, casein and whey could generate different effects on metabolism [51]. In an intervention study, Hopped et al. revealed that eight-year-old children who received casein experienced an increase in serum IGF-1. In contrast, those taking whey experienced an increase in fasting insulin [51]. Moreover, Lönnerdal et al. found that adding osteopontin to infant formula could reduce the level of branched-chain amino acids in the plasma of formula-fed infants [20]. The mechanisms of how proteins regulate infant growth are still unclear, and further studies are needed.

Since α_s-1_ casein is recognized as a small part of casein and has received little attention in the literature, our knowledge about the impact of α_s-1_ casein on the growth of infants is limited. However, an animal experiment showed that α-casein deficiency led to a permanent reduction in the body size of mice [52], indicating that α-casein in milk may play a critical role in growth. Our study found that the concentration of α_s-1_ casein in HM was inversely associated with the weight-for-age Z-score of infants at one month and six months postpartum. The underlying mechanism is unclear. Considering that the association of α_s-1_ casein in relation to the length of infants was insignificant, α_s-1_ casein might be a regulatory factor for the weight of infants.

Our study revealed that some factors from the mothers and delivery are related to the concentrations of milk proteins. Previous studies suggested that maternal age is an impact factor of HM components and that an older age might be related to lower protein concentrations in some populations [36,53]. The present study observed that women aged 30 years and above had a relatively lower concentration of total protein in the colostrum but a higher concentration in mature milk. The longitudinal changes of total protein tended to be influenced by maternal age (*p*-interaction 0.067). Moreover, the longitudinal changes in osteopontin were also impacted by maternal age. Aging may cause changes in the mammary glands. However, the effects of maternal age on milk protein remain controversial [40,54,55]. Further research is needed to investigate the underlying mechanism of this association. Second, our study found that women who had a Cesarean delivery had a lower concentration of total casein and β-casein in colostrum, but slower decreases with the progression of lactation were observed for total casein, β-casein, and lactoferrin among women who had a Cesarean delivery. This may be partly because the hormonal activity induced by labor pain and uterine contractions were different between women who gave Cesarean and vaginal delivery [54]. Nissen et al. revealed different patterns of oxytocin and prolactin release during breastfeeding on day 2 postpartum in women who delivered vaginally or via Cesarean section [56]. Moreover, Cesarean delivery was reported to be related to a late onset of lactation [57], which may influence the concentration of casein in colostrum and delay the decline across lactation. Third, the present study found that higher education was related to a higher concentration of total protein in early mature milk, higher α-lactalbumin in colostrum, and higher osteopontin in mature milk. The longitudinal changes of α-lactalbumin were impacted by maternal education and income. This result might be because Chinese lactating women with higher education and income had higher intakes of animal-sourced food, including dairy products, meat, poultry, fish, and eggs [58], which might further promote the secretion of proteins [59,60,61]. Lastly, although the pre-gestational BMI of mothers did not impact the longitudinal changes of milk proteins in the study population, we observed that lactating women with higher BMIs had higher concentrations of lactoferrin and α_s-1_ casein in early mature milk. The underlying mechanism of this relationship is still unclear.

The present study has several limitations. First, the concentration of total protein in milk was measured by the Bradford method instead of the Kjeldahl method, as the milk sample volumes were limited. Second, the proteins and the free amino acids in HM are important for infant health. Due to the restriction of resources and funding, the present study only investigated major proteins in HM, and only about half of the samples were tested for all indices in this study. Third, the present study did not take maternal diet into consideration, which might have an impact on milk composition. Fourth, previous studies suggested that both the concentration of proteins in HM and daily protein intake were associated with the growth of infants [62]. However, the present study did not record the amount of HM consumed by the infants. Further studies should be conducted to estimate the association between the daily intake of protein and the growth of term infants.

## 5. Conclusions

The present study provided comprehensive information on proteins in milk from Chinese women. It revealed that the concentration of most major proteins in milk decreased over the course of lactation. The concentrations and longitudinal changes of milk proteins were impacted by factors related to the mothers and delivery. The concentration of α_s-1_ casein in milk was inversely associated with the weights of the infants. These results contribute to our knowledge of human milk composition. Future studies including larger sample sizes and longer follow-up periods are necessary. Furthermore, prospective studies with longer follow-up periods are needed to characterize the impact of milk proteins, especially caseins, on infant growth.

## Figures and Tables

**Table 1:** Baseline characteristics of lactating mothers and corresponding infants ^a^.

Variables	All Participants	Subgroup	*p*
Number of participants	105	51	
Maternal age (years)			>0.999
Below 30	58.1	56.9	
30 and above	41.9	43.1	
Education			0.261
Middle school and below	38.1	49.0	
College and above	61.9	51.0	
Per capita household income (RMB/month)			0.587
4000 and below	35.2	41.2	
Above 4000	64.8	58.8	
Pre-gestational body mass index (kg/m^2^) ^b^			0.807
<20.7	46.7	43.1	
≥20.7	53.3	56.9	
Delivery mode			0.195
Cesarean delivery	25.7	37.3	
Vaginal delivery	74.3	62.7	
Parity			0.767
First birth	78.1	74.5	
Others	21.9	25.5	
Infant gender			>0.999
Female	40.0	39.2	
Male	60.0	60.8	

Variables are presented as percentages. Differences in baseline characteristics between all participants and those in the subgroup were compared using Chi-square tests. ^a^ The concentrations of total protein and α-lactalbumin in the milk samples from all the participants were measured, while the concentrations of other indices were only tested for 51 participants. ^b^ Pre-gestational body mass index was converted into a categorical variable based on the median (<20.7 or ≥20.7 kg/m^2^).

**Table 2:** Concentration of human milk proteins during the first six months of lactation ^a^.

	1–5 Days (t1)	8–14 Days (t2)	1 Month (t3)	6 Months (t4)	*p*-Trend	*p*(t2 vs. t1)	*p*(t3 vs. t2)	*p*(t4 vs. t3)
Number of samples (overall participants)	103	105	105	98				
Total protein (mg/100 mL)	1666.2 (447.7)	1545.2 (416.7)	1368.3 (406.3)	993.9 (335.0)	<0.001	0.013	<0.001	<0.001
α-lactalbumin (mg/100 mL)	327.9 (63.4)	333.9 (58.5)	310.9 (57.3)	209.0 (52.6)	<0.001	0.326	<0.001	<0.001
Number of samples (subgroup)	49	51	51	46				
Lactoferrin (mg/100 mL) ^b^	298.5 (154.1)	189.5 (81.6)	114.9 (45.4)	70.4 (27.4)	<0.001	<0.001	<0.001	<0.001
Osteopontin (mg/100 mL)	71.8 (30.3)	58.6 (15.1)	45.0 (14.8)	23.6 (12.3)	<0.001	0.001	<0.001	<0.001
Total casein (mg/100 mL)	697.1 (191.0)	632.2 (139.8)	541.5 (122.7)	373.9 (120.8)	<0.001	0.007	<0.001	<0.001
β-casein (mg/100 mL)	520.9 (147.8)	484.4 (117.9)	439.4 (103.3)	315.4 (101.5)	<0.001	0.050	0.039	<0.001
α_s-1_ casein (mg/100 mL)	125.4 (44.3)	104.4 (35.6)	69.2 (20.9)	37.9 (19.4)	<0.001	0.001	<0.001	<0.001
κ-casein (mg/100 mL)	50.8 (15.9)	43.4 (10.4)	32.8 (7.9)	20.6 (6.3)	<0.001	<0.001	<0.001	<0.001

Values are presented as the means and standard deviations. Linear mixed effects models were used to estimate the longitudinal changes in concentrations of human milk proteins across lactation periods with postpartum time (weeks) as a fixed effect and the participants as a random intercept. Differences in the concentrations of human milk proteins between surveys were analyzed using one-way repeated measures ANOVA tests. ^a^ The concentrations of total protein and α-lactalbumin in the milk samples from all the participants were measured, while the concentrations of other indices were only tested for 51 participants in the subgroup. ^b^ The values of lactoferrin concentration in milk were transformed to the log scale before analysis.

**Table 3:** Anthropometric indices of infants at one month and six months postpartum.

	One Month Postpartum	Six Months Postpartum	*p*
Length-for-age Z-score	0.38 (0.93)	0.63 (1.31)	0.036
Weight-for-age Z-score	0.47 (1.06)	0.49 (1.17)	0.841
Weight-for-length Z-score	0.16 (1.47)	0.28 (1.16)	0.453

Values are presented as the means and standard deviations. Differences in Z-scores between surveys were analyzed by one-way repeated measures ANOVA tests.

## Data Availability

The datasets analyzed in this study are available from the corresponding author upon reasonable request.

## Supplementary Materials

The following are available online at https://www.mdpi.com/article/10.3390/nu13051476/s1. Table S1: Internal standards used in protein quantification. Table S2: Quality control in the quantification of milk proteins. Table S3: Concentrations of human milk proteins across baseline characteristics.
