# Supplementary material for: Longitudinal Changes in the Concentration of Major Human Milk Proteins in the First Six Months of Lactation and Their Effects on Infant Growth
Source: Nutrients. 2021 Apr 27;13(5):1476. doi: 10.3390/nu13051476 (PMC8147063; doi:10.3390/nu13051476)
Supplement: Supplementary file 1 [file nutrients-13-01476-s001.zip › TableS1.pdf]

Table S1. Internal standards used in protein quantification.

Protein	Amino acid sequence	Concentration (μg/mL)
α-lactalbumin	CELSQLLK	20
Lactoferrin	VPSHAVVAR	10
β-casein	VMPVLK	20
α_{s-1} casein	NNVMLQW	20
κ-casein	QYLPNSHPPTVVR	20
Osteopontin	GDSVVYGLR	10

Internal standards were purchased from ChinaPeptides Co. Ltd (Shanghai, China).
