# Supplementary material for: Longitudinal Changes in the Concentration of Major Human Milk Proteins in the First Six Months of Lactation and Their Effects on Infant Growth
Source: Nutrients. 2021 Apr 27;13(5):1476. doi: 10.3390/nu13051476 (PMC8147063; doi:10.3390/nu13051476)
Supplement: Supplementary file 1 [file nutrients-13-01476-s001.zip › TableS2.pdf]

Table S2. Quality control in the quantification of milk proteins.

Protein	Number of tests	Coefficient of variation (%)
α-lactalbumin	14	9.0
Lactoferrin	8	4.6
β-casein	8	6.6
$\alpha_{s\text{-}1}$ casein	8	5.5
κ-casein	8	6.6
Osteopontin	6	9.3

When testing the milk samples from the present study, another human milk sample that was previously collected was tested simultaneously every day. The coefficients of variation of protein concentrations in the identical milk sample were presented.
