# Supplementary material for: Longitudinal Changes in the Concentration of Major Human Milk Proteins in the First Six Months of Lactation and Their Effects on Infant Growth
Source: Nutrients. 2021 Apr 27;13(5):1476. doi: 10.3390/nu13051476 (PMC8147063; doi:10.3390/nu13051476)
Supplement: Supplementary file 1 [file nutrients-13-01476-s001.zip › TableS3.pdf]

Table S3. Concentrations of human milk proteins across baseline characteristics.

		1–5 days	8–14 days	1 month	6 months	*p*-interaction
			**Total protein**			
Maternal age (years)	Below 30	1738.3(422.6)	1496.7(402.2)	1339.7(453.2)	930.4(338.4)*	0.067
	30 and above	1565.7(467.0)	1612.4(431.5)	1407.9(331.4)	1078.5(314.7)	
Education	Below college	1663.5(501.9)	1501.2(318.2)	1268.2(399.6)*	943.9(297.5)	0.738
	College and above	1668.0(413.8)	1572.2(467.4)	1429.8(401.0)	1022.9(354.0)	
Per capita household income (RMB/month)	4000 and below	1636.7(504.7)	1581.6(387.2)	1311.1(402.5)	939.8(278.5)	0.445
	Above 4000	1681.4(418.5)	1525.3(433.4)	1399.4(407.9)	1020.1(358.2)	
Pre-gestational body mass index (kg/m^2)	<20.7	1651.9(478.9)	1513.7(445.7)	1352.7(413.5)	991.4(378.9)	0.748
	≥20.7	1678.3(423.7)	1572.7(391.5)	1381.9(403.1)	995.9(298.2)	
Mode of delivery	Cesarean delivery	1631.3(507.7)	1587.7(320.1)	1348.1(319.5)	1089.9(353.7)	0.149
	Vaginal delivery	1678.0(428.5)	1530.4(446.1)	1375.3(433.9)	961.0(324.4)	
Parity	First parity	1681.3(424.6)	1520.1(421.2)	1372.3(413.2)	982.3(338.0)	0.788
	Others	1610.7(531.5)	1634.5(396.0)	1353.9(388.8)	1033.9(328.8)	
Infant gender	Female	1617.7(486.9)	1565.2(445.0)	1386.3(431.7)	978.7(277.5)	0.938
	Male	1697.0(422.0)	1531.8(399.8)	1356.3(391.5)	1003.9(370.1)	
			**α-lactalbumin**			
Maternal age (years)	Below 30	325.6(67.2)	327.2(65.3)	310.7(60.2)	210.1(58.0)	0.390
	30 and above	331.2(58.3)	343.2(46.7)	311.1(53.8)	207.7(45.0)	
Education	Below college	309.3(68.6)*	320.2(63.7)	305.7(52.3)	214.6(54.2)	0.004
	College and above	339.7(57.3)	342.3(53.9)	314.1(60.4)	205.8(51.8)	
Per capita household income (RMB/month)	4000 and below	312.8(72.1)	327.0(67.5)	307.0(53.9)	222.6(57.1)	0.002
	Above 4000	335.7(57.4)	337.7(53.2)	313.0(59.3)	202.5(49.3)	
Pre-gestational body mass	<20.7	324.1(65.1)	329.9(62.1)	308.9(66.5)	205.0(53.3)	0.759

index (kg/m²)						
	≥20.7	331.1(62.3)	337.4(55.6)	312.6(48.4)	212.4(52.2)	
Mode of delivery	Cesarean delivery	327.6(65.8)	351.4(62.9)	300.9(46.6)	220.7(62.4)	0.378
	Vaginal delivery	328.0(63.0)	327.8(56.1)	314.3(60.5)	205.1(48.6)	
Parity	First parity	328.8(62.5)	336.3(59.0)	313.7(59.7)	207.5(53.3)	0.173
	Others	324.7(67.9)	325.4(57.5)	301.0(47.8)	214.3(50.8)	
Infant gender	Female	325.6(63.5)	337.9(49.8)	308.3(58.3)	203.6(46.9)	0.291
	Male	329.4(63.8)	331.2(63.9)	312.6(57.0)	212.6(56.1)	
**Lactoferrin**						
Maternal age (years)	Below 30	281.3(105.0)	169.1(55.7)	109.2(42.0)	64.0(18.5)	0.746
	30 and above	321.5(202.8)	216.2(101.9)	122.4(49.5)	78.0(34.2)	
Education	Below college	286.4(110.8)	197.6(81.8)	125.9(49.1)	68.5(15.9)	0.781
	College and above	311.2(190.8)	181.6(82.2)	104.3(39.6)	72.3(35.7)	
Per capita household income (RMB/month)	4000 and below	253.6(99.2)	184.6(82.6)	116.6(47.7)	70.2(14.9)	0.211
	Above 4000	327.0(176.1)	192.9(82.1)	113.7(44.5)	70.6(33.8)	
Pre-gestational body mass index (kg/m²)	<20.7	278.6(130.4)	168.5(68.3)	100.2(35.5)*	66.3(21.4)	0.508
	≥20.7	312.3(169.4)	205.4(88.2)	126.1(49.3)	73.3(31.0)	
Mode of delivery	Cesarean delivery	284.0(145.9)	195.7(100.1)	118.6(51.6)	87.7(33.3)***	0.024
	Vaginal delivery	307.0(160.4)	185.7(69.9)	112.7(42.0)	61.2(18.4)	
Parity	First parity	304.4(124.0)	179.1(64.0)	113.1(43.0)	65.8(22.9)	0.150
	Others	280.6(229.9)	219.6(117.5)	120.3(53.2)	82.0(34.8)	
Infant gender	Female	315.3(193.4)	195.1(78.4)	117.9(38.3)	79.3(32.6)	0.435
	Male	288.8(128.6)	185.8(84.7)	113.0(49.9)	65.7(23.4)	
**Osteopontin**						
Maternal age (years)	Below 30	77.3(27.6)	59.5(15.0)	47.9(12.4)	19.8(12.8)*	0.018
	30 and above	64.4(32.8)	57.5(15.4)	41.1(16.9)	28.0(10.3)	

Education	Below college	70.6(31.9)	56.6(15.1)	47.5(13.0)	18.8(13.2)**	0.198
	College and above	73.0(29.2)	60.6(15.0)	42.5(16.2)	28.3(9.2)	
Per capita household income (RMB/month)	4000 and below	61.3(31.4)	54.9(18.1)	45.6(13.9)	21.3(12.8)	0.415
	Above 4000	78.4(28.2)	61.3(12.1)	44.5(15.6)	25.2(11.9)	
Pre-gestational body mass index (kg/m^2)	<20.7	70.7(23.3)	58.3(13.7)	42.8(12.2)	23.9(11.3)	0.672
	≥20.7	72.5(34.7)	58.9(16.2)	46.6(16.5)	23.3(13.2)	
Mode of delivery	Cesarean delivery	65.3(26.3)	58.4(13.8)	44.1(15.3)	27.5(11.8)	0.133
	Vaginal delivery	75.5(32.3)	58.8(16.0)	45.5(14.7)	21.5(12.2)	
Parity	First parity	73.6(26.7)	59.9(13.1)	49.1(11.5)***	23.6(12.4)	0.240
	Others	66.2(40.4)	55.0(19.8)	32.8(17.0)	23.5(12.5)	
Infant gender	Female	71.1(28.3)	58.3(15.4)	40.5(17.9)	26.8(12.8)	0.279
	Male	72.1(31.9)	58.9(15.1)	47.8(11.8)	21.8(11.8)	
**Total casein**						
Maternal age (years)	Below 30	712.9(182.9)	604.6(129.3)	522.7(83.3)	341.5(132.7)*	0.275
	30 and above	676.1(204.0)	668.7(147.6)	566.3(159.6)	412.5(93.8)	
Education	Below college	698.5(216.4)	620.4(135.0)	554.3(130.2)	360.4(110.4)	0.693
	College and above	695.7(165.2)	643.6(146.1)	529.1(116.2)	387.5(131.4)	
Per capita household income (RMB/month)	4000 and below	675.6(236.6)	614.4(131.4)	553.6(134.8)	372.2(127.0)	0.565
	Above 4000	710.8(158.7)	644.7(146.3)	533.0(115.1)	375.1(118.7)	
Pre-gestational body mass index (kg/m^2)	<20.7	689.1(181.5)	602.2(123.5)	505.3(97.8)	356.0(144.6)	0.898
	≥20.7	702.7(200.3)	655.0(149.1)	568.9(133.8)	386.5(101.9)	
Mode of delivery	Cesarean delivery	624.1(198.1)*	630.1(169.7)	532.1(165.1)	419.2(141.5)	0.013
	Vaginal delivery	739.5(176.4)	633.5(121.6)	547.0(91.5)	349.8(102.7)	
Parity	First parity	706.5(190.0)	628.2(110.3)	545.1(88.7)	372.7(118.5)	0.713

	Others	668.3(199.7)	643.9(209.1)	530.9(195.8)	377.0(131.3)	
Infant gender	Female	687.3(214.2)	652.9(144.2)	504.7(111.2)	391.5(110.7)	0.578
	Male	702.8(179.8)	618.9(137.6)	565.2(125.6)	364.5(126.6)	
			**β-casein**			
Maternal age (years)	Below 30	533.3(142.0)	465.6(95.8)	423.2(65.8)	290.0(111.4)	0.252
	30 and above	504.3(157.2)	509.2(140.4)	460.7(137.1)	345.7(80.7)	
Education	Below college	518.5(167.1)	472.5(118.5)	449.9(110.7)	305.7(90.0)	0.822
	College and above	523.4(128.3)	495.9(118.5)	429.3(96.8)	325.1(113.0)	
Per capita household income (RMB/month)	4000 and below	504.7(174.6)	462.7(117.4)	449.5(115.2)	310.7(104.0)	0.619
	Above 4000	531.1(130.3)	499.6(117.8)	432.3(95.6)	318.7(101.5)	
Pre-gestational body mass index (kg/m^2)	<20.7	522.1(137.6)	463.6(95.1)	410.5(81.4)	300.2(122.5)	0.898
	≥20.7	520.1(156.8)	500.2(132.0)	461.3(113.8)	326.1(84.5)	
Mode of delivery	Cesarean delivery	465.9(148.8)*	480.0(146.9)	432.4(141.5)	351.7(121.3)	0.013
	Vaginal delivery	552.8(139.8)	487.1(99.3)	443.6(74.5)	296.1(85.2)	
Parity	First parity	526.7(148.2)	482.2(87.9)	441.1(74.5)	315.1(100.2)	0.788
	Others	503.1(151.7)	490.9(184.5)	434.4(165.4)	316.3(108.9)	
Infant gender	Female	507.5(170.4)	495.8(117.9)	404.8(95.8)	325.1(92.1)	0.551
	Male	528.6(135.4)	477.1(119.2)	461.8(103.2)	310.2(107.3)	
			**α_{s-1} casein**			
Maternal age (years)	Below 30	125.4(45.4)	94.7(33.5)*	65.3(18.1)	31.4(20.0)*	0.736
	30 and above	125.4(44.0)	117.3(34.8)	74.4(23.5)	45.7(15.9)	
Education	Below college	128.2(48.4)	105.6(36.9)	71.5(21.6)	35.1(19.5)	0.487
	College and above	122.4(40.5)	103.4(35.0)	67.1(20.4)	40.6(19.4)	
Per capita household income (RMB/month)	4000 and below	121.2(54.9)	108.2(36.6)	70.5(20.7)	39.0(21.2)	0.631
	Above 4000	128.1(37.0)	101.8(35.2)	68.3(21.4)	37.1(18.5)	

Pre-gestational body mass index (kg/m²)	<20.7	115.8(43.1)	94.4(32.4)	61.7(17.1)*	34.4(19.4)	0.371
	≥20.7	132.1(44.7)	112.1(36.5)	74.9(21.9)	40.3(19.5)	
Mode of delivery	Cesarean delivery	109.7(40.7)	106.2(41.0)	68.0(22.7)	43.9(20.7)	0.082
	Vaginal delivery	134.5(44.4)	103.4(32.6)	70.0(20.1)	34.7(18.3)	
Parity	First parity	128.4(45.5)	103.0(32.7)	70.6(18.9)	38.0(17.9)	0.745
	Others	116.0(41.1)	108.8(44.2)	65.2(26.4)	37.7(23.8)	
Infant gender	Female	129.6(42.8)	111.0(32.8)	67.2(16.5)	43.5(17.4)	0.881
	Male	123.0(45.7)	100.2(37.2)	70.6(23.5)	34.9(20.1)	
**κ-casein**						
Maternal age (years)	Below 30	54.2(15.4)	44.3(10.5)	34.1(7.3)	20.1(6.5)	0.162
	30 and above	46.4(15.8)	42.2(10.4)	31.1(8.4)	21.2(6.3)	
Education	Below college	51.8(17.9)	42.4(11.1)	33.0(6.7)	19.5(5.6)	0.645
	College and above	49.9(13.9)	44.3(9.8)	32.7(9.0)	21.7(7.0)	
Per capita household income (RMB/month)	4000 and below	49.7(18.7)	43.5(10.7)	33.7(7.2)	22.5(6.4)	0.186
	Above 4000	51.5(14.2)	43.3(10.3)	32.3(8.4)	19.2(6.1)	
Pre-gestational body mass index (kg/m²)	<20.7	51.3(14.5)	44.2(7.7)	33.0(8.2)	21.4(6.6)	0.825
	≥20.7	50.6(17.1)	42.7(12.1)	32.7(7.8)	20.1(6.2)	
Mode of delivery	Cesarean delivery	48.6(18.4)	44.0(11.4)	31.7(5.9)	23.6(6.8)*	0.083
	Vaginal delivery	52.2(14.5)	43.0(9.9)	33.5(8.9)	19.0(5.6)	
Parity	First parity	51.4(16.7)	43.1(9.9)	33.4(7.9)	19.7(6.3)	0.291
	Others	49.2(13.8)	44.2(12.2)	31.3(8.1)	22.9(6.0)	
Infant gender	Female	50.2(15.5)	46.1(9.8)	32.8(8.1)	22.8(8.0)	0.592
	Male	51.2(16.4)	41.6(10.5)	32.9(7.9)	19.4(5.0)	

* $p < 0.05$, ** $p < 0.01$, *** $p < 0.001$. Values were presented as means and standard deviations. Concentrations of milk proteins were expressed as g/100 mL. Pre-gestational body mass index was converted into a categorical variable based on the median (<20.7 or ≥20.7 kg/m²). The overall effects of baseline characteristics on the concentrations of milk proteins were investigated with the linear mixed effects models. Each model included the fixed effects of postpartum time (weeks), a predictor (maternal age, education, household income, pre-gestational body

mass index, mode of delivery, parity, and gender of infant), and an interaction item for postpartum time and the predictor, as well as a random intercept for each participant. Differences in milk protein concentrations across baseline characteristics at each time point were compared using Student's t-test. The values of lactoferrin concentration in milk were transformed to the log scale before analysis.
